# A simple computational principle predicts vocal adaptation dynamics across age and error size

**DOI:** 10.3389/fnint.2014.00075

**Published:** 2014-09-29

**Authors:** Conor W. Kelly, Samuel J. Sober

**Affiliations:** Department of Biology, Emory UniversityAtlanta, GA, USA

**Keywords:** sensorimotor adaptation, vocal learning, error correction, age-related changes, songbird, sensorimotor integration, pitch-shift

## Abstract

The brain uses sensory feedback to correct errors in behavior. Songbirds and humans acquire vocal behaviors by imitating the sounds produced by adults and rely on auditory feedback to correct vocal errors throughout their lifetimes. In both birds and humans, acoustic variability decreases steadily with age following the acquisition of vocal behavior. Prior studies in adults have shown that while sensory errors that fall within the limits of vocal variability evoke robust motor corrections, larger errors do not induce learning. Although such results suggest that younger animals, which have greater vocal variability, might correct large errors more readily than older individuals, it is unknown whether age-dependent changes in variability are accompanied by changes in the speed or magnitude of vocal error correction. We tested the hypothesis that auditory errors evoke greater vocal changes in younger animals and that a common computation determines how sensory information drives motor learning across different ages and error sizes. Consistent with our hypothesis, we found that in songbirds the speed and extent of error correction changes dramatically with age and that age-dependent differences in learning were predicted by a model in which the overlap between sensory errors and the distribution of prior sensory feedback determines the dynamics of adaptation. Our results suggest that the brain employs a simple and robust computational principle to calibrate the rate and magnitude of vocal adaptation across age-dependent changes in behavioral performance and in response to different sensory errors.

## Introduction

Both humans and songbirds acquire vocal behavior by a process of imitation, employing sensory feedback to reduce initially large errors until vocalizations approach a sensory target, and the brain continues to use auditory feedback to correct vocal errors throughout adulthood (Houde and Jordan, 1998; Hoffmann and Sober, 2014). After acquiring vocal behavior, vocal variability decreases steadily with age in both humans and songbirds (Lee et al., 1999; Deregnaucourt et al., 2005). In songbirds, a basal ganglia-thalamo-cortical pathway is critical for both driving adaptive motor changes during learning and for generating vocal motor variability (Kao et al., 2005; Olveczky et al., 2005; Andalman and Fee, 2009; Warren et al., 2011), suggesting a close link between variability and vocal learning. However, it is unknown whether age-dependent decreases in variability are associated with changes in the dynamics of vocal error correction (or “sensorimotor adaptation”).

When sensory feedback signals that an error has occurred, the brain must compute how much to modify behavior. Prior studies suggest that the brain uses the variability of prior motor production to compute whether or not to modify behavior after experiencing a sensory error. In principle, the brain could correct apparent sensory errors when they overlap with the probability distribution of previously experienced sensory feedback but ignore larger errors. In adult songbirds, larger auditory errors evoke smaller vocal changes than smaller errors, and the magnitude of learning approaches zero when auditory feedback falls outside of the baseline probability distribution (Sober and Brainard, 2012), consistent with studies in other systems showing that adults exhibit reduced learning to larger errors (Robinson et al., 2003; Wei and Kording, 2009). However, because these studies in adults did not dissociate error probability from error size it is unclear whether learning differences result from overlap with prior sensory experience *per se* or from absolute error magnitude. Additionally, it is unknown whether this principle predicts learning dynamics in young animals or can account for age-dependent differences in error correction.

**Figure 1:**
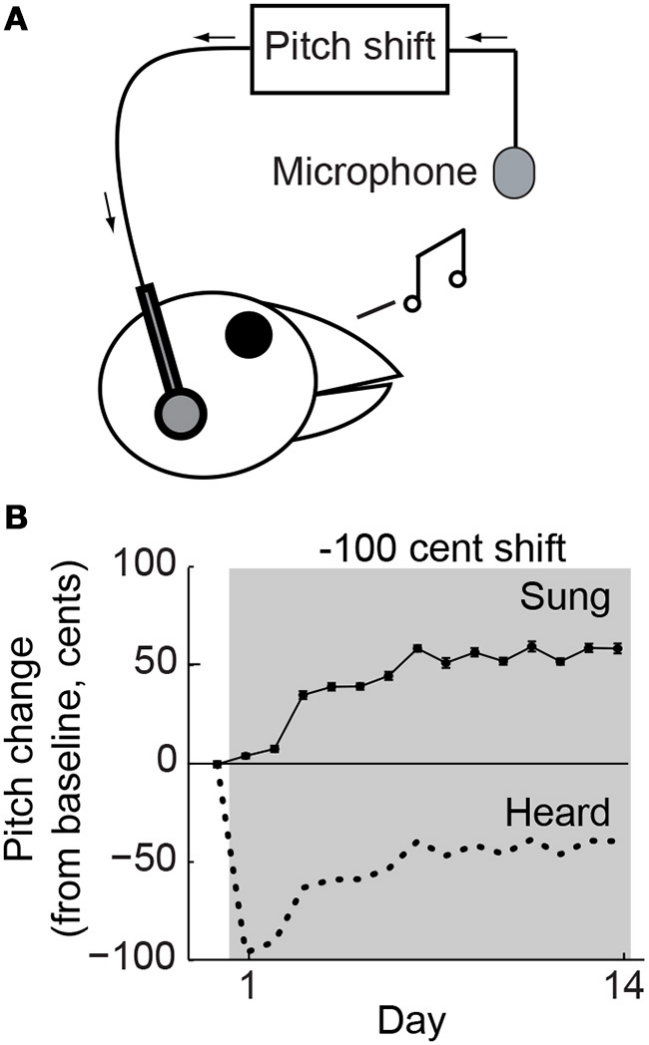
**Behavioral paradigm. (A)** Online shifts in the pitch of auditory feedback were provided by miniaturized headphones. **(B)** Changes in mean vocal pitch in response to a 100-cent (1 semitone; see Methods) downward shift in the pitch of auditory feedback for a single representative experiment. Pitch shifts were always applied relative to current vocal production so that the difference between sung (solid line) and heard (dotted line) pitch was constant. The upward change in vocal pitch brought the heard pitch closer to the baseline value, reducing the experienced error.

We addressed these questions by comparing vocal plasticity in songbirds across different error sizes shortly after song acquisition (~100 days old) and later in adulthood (>190 days old). We investigated whether vocal error correction in response to auditory perturbations (Figure 1) changes during age-dependent reductions in vocal variability. Further analysis exploited age-dependent differences in variability to dissociate variability from the size of sensory errors and test the hypothesis that distribution overlap, rather than error size, is the best predictor of adaptation dynamics. We predicted that the same sensory error would evoke greater motor changes in younger adults. Additionally, we hypothesized that a mathematical model based on distribution overlap would predict differences in adaptation across both age and different error magnitudes. Such results would indicate that although the dynamics of adaptation may differ across age and error size, the relationship between adaptation dynamics and the statistics of sensorimotor experience is constant.

## Materials and methods

Online sound-processing hardware was used to shift the fundamental frequency (which we refer to here as “pitch”) of auditory feedback (Figure 1A), which was played back through lightweight headphones with a typical loop delay of <10 ms, as described previously (Hoffmann et al., 2012). As in our prior studies, the headphones shielded the ears from direct airborne sound transmission and served to replace natural auditory feedback with the manipulated version. Furthermore, pitch shifts were always performed relative to the bird's current acoustic output. Pitch shifts therefore created a constant offset between the sung (Figure 1B, solid line) and heard (Figure 1B, dotted line) pitch so that compensatory vocal changes brought the heard pitch closer to the baseline value (Figure 1B, upward trend in dotted line), thereby reducing the experienced error.

To quantify how the dynamics of sensorimotor adaptation change with age, we collected data from two groups of male Bengalese finches: “young-adults” (*n* = 13; mean age 106 days post-hatch, or “dph,” range 92–118 at pitch shift onset) and “adults” (*n* = 11; mean age 377 dph, range 198–1083 at shift onset). After the headphones were attached and birds acclimated to the apparatus, each bird sang with unshifted feedback during a baseline period of 2–8 days. Following the baseline period, feedback to the headphones was shifted in pitch by ±100 cents (up or down one semitone), ±300 cents (up or down three semitones), or ±600 cents (up or down six semitones). The shifted feedback was maintained for 14 days. Data from a subset of the adult birds have been presented previously as part of a separate analysis (Sober and Brainard, 2012); all data from the young-adult birds exposed to ±100, ±300, and ±600 cent shifts are presented here for the first time. In no cases were young-adult songbirds re-tested as adults (i.e., the young-adult and adult groups are non-overlapping populations). Note that Bengalese finches have a typical lifespan of >5 years. All procedures were approved by Emory University Institutional Animal Care and Use Committee.

### Acoustic analysis

To quantify changes in vocal pitch, we measured pitch changes at particular times (“spectral frames”) within each iteration of song syllables, as described previously (Sober and Brainard, 2009). Birds produced a median of 6 distinct syllables (range, 3–11) with quantifiable pitch. Iterations of distinct syllables were labeled by visual inspection of spectrograms. As in prior studies (Sober and Brainard, 2009; Hoffmann and Sober, 2014), we quantified pitch for songs produced between 10:00 a.m. and 12:00 p.m. (we have previously demonstrated that learning in this paradigm does not vary significantly based on the time of day sampled; Sober and Brainard, 2009). For a small number of days during which data were not available within that time period, available song files from the same day but outside of the time window were quantified; this contributed less than 10% of the data analyzed.

To quantify the changes in vocal pitch from baseline values, pitch measurements in Hz were converted to units of cents. An interval of 100 cents is equivalent to one semitone, and there are 12 semitones (1200 cents) in an octave. A pitch difference in units of cents is computed from the difference in frequency as follows:

$$p=1200\log_{2}\frac{h_{x}}{b}$$

where *p* is the change in pitch of the spectral frame (in cents), *h_x_* is the pitch (in Hz) of the spectral frame of the syllable, and *b* is the average pitch (in Hz) of the spectral frame of the syllable across the baseline period.

### Model fitting: standard exponential model

The following model (“standard exponential model”) was fit to pitch data to quantify the timescale of sensorimotor adaptation and the fraction of the sensory error compensated at equilibrium:

(1) $$p(t)=-cE(1-e^{-\frac{t}{\tau }})$$

where *p(t)* is the change in mean pitch (in cents) of song syllables on post-shift day *t* and *E* is the experimentally-induced pitch shift (in cents). Free parameters *c* and τ represent the fraction of the pitch perturbation compensated at equilibrium and the time constant of adaptation, respectively. Permutation tests were used to test for significant differences in *c* and τ across experimental conditions as described previously (Sober and Brainard, 2012). Additionally, we fit an alternate version of Equation 1 in which adaptation is quantified not in units of time but in the number of syllables produced:

(2) $$p(n)=-cE(1-e^{-\frac{n}{\tau }})$$

where *n* represents the total number of song syllables produced since the onset of the pitch shift and τ represents the time constant of learning, expressed here in terms of number of song syllables. To fit this model to the data, we estimated total daily song syllable production during each experiment by measuring the total number of song syllables produced on 2–5 representative days during the shift epoch.

### Model fitting: prior overlap model

We also present a model (“prior overlap model”) in which the dynamics of sensorimotor adaptation are predicted by the overlap between the bird's baseline pitch distribution and the distribution experienced following the introduction of the shift. Overlap (ν) was calculated using the following equation:

$$\nu =\frac{\int r(x)r\left(x+E\right)dx}{\int r{\left(x\right)}^{2}dx}$$

where *r(x)* is the probability density function of pitches produced during the baseline condition and *E* is the induced pitch shift perturbation (Sober and Brainard, 2012). We then used overlap to predict the values of parameters *c* and τ in Equation 1, as follows:

$$\begin{array}{l} c=\alpha_{c}\nu^{\rho }\\ \tau =\alpha_{\tau }\nu^{\rho } \end{array}$$

where free parameters α_*c*_, α_τ_, and ρ quantify the relationship between overlap and the speed and completeness of sensorimotor adaptation. We selected power laws because they use a small number of parameters to describe the relationship between overlap and adaptation dynamics in data drawn from adult songbirds, allowing us to easily compute whether data from young-adult animals obey the same relationship, as described below. The full prior overlap model can then be written as:

(3) $$p\left(t\right)=-\alpha_{c}\nu^{\rho }E(1-e^{\frac{-t}{\alpha_{\tau }\nu^{\rho }}})$$

### Model selection

To compare the predictive power of the two models described above, we expanded our dataset by combining the data from ±100, ±300, and ±600 cent shifts in young-adults and adults (*n* = 24 birds, see above) with additional datasets from 9 adults exposed to four other shift sizes (±50 and ±150 cents) that were collected as part of a separate study (Sober and Brainard, 2012). First, we used an *F* test to ask whether the prior overlap model (Equation 3) has greater predictive power than the standard exponential model (Equation 1). Note that the two models are nested: setting parameter ρ in Equation 3 equal to zero reduces the prior overlap model to the standard exponential model. To quantify whether the prior overlap model (which has 3 free parameters) has significantly greater predictive power than the standard exponential model (which has 2), we therefore performed an *F* test on the “extra sum of squares” obtained by including the additional free parameter in the overlap model (Draper and Smith, 1998) when the models are fit to the expanded dataset that includes all animal ages and shift sizes.

Second, we asked how well the prior overlap model predicts sensorimotor adaptation across age. To ask whether parameters α_*c*_, α_τ_, and ρ differ between young-adult versus adult songbirds, we performed *F* tests on the extra sum of squares obtained by fitting parameters in Equation 3 separately to data from young-adult subjects. For example, to ask whether the parameter α_*c*_ differed significantly, we fit one model in which α_*c*_, α_τ_, and ρ were all fit using data from both ages, and another in which a different value of α_*c*_ was fit to the young-adult data. We then tested whether the additional variance accounted for in the second model (which had four free parameters) was a significant (*p* < 0.05) improvement over the predictive power of the first model (which had three free parameters). Significant differences for α_τ_ and ρ were computed by the same technique.

## Results

**Figure 2:**
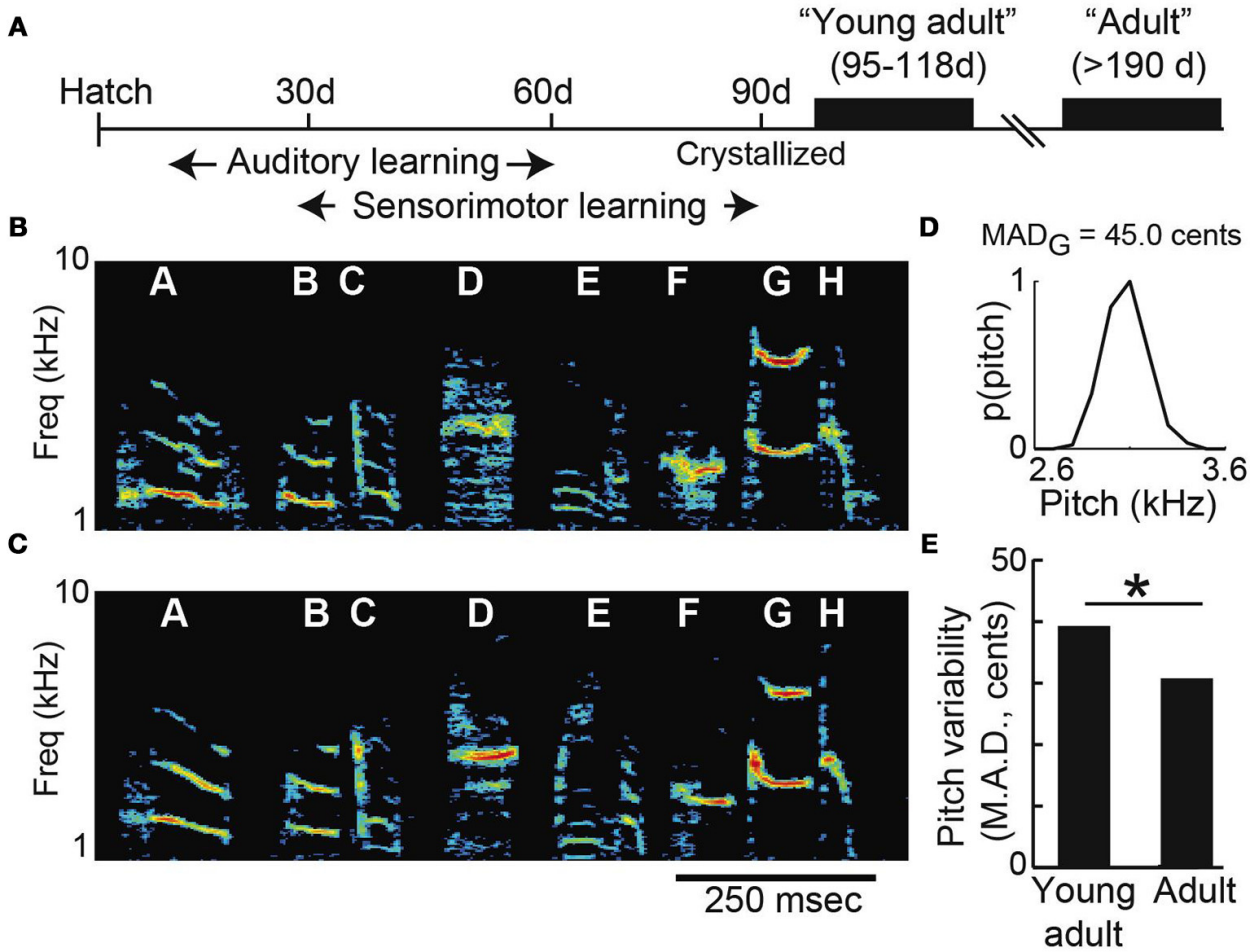
**Vocal variability decreases across early adulthood. (A)** Timeline of song development. Songbirds learn their songs by a process of vocal imitation. By 90 days of age, the basic acoustic elements of song have been acquired (“crystallized”). Spectrograms show songs recorded from the same Bengalese finch as a young-adult (**B**; 103 days of age) and later as an adult (**C**; 353 days). Spectrograms show the power (color scale) at each acoustic frequency as a function of time. **(D)** The pitch of syllable “G” in **(B)** is variable, with a median absolute deviation (MAD) of 45.0 cents (see Methods). **(E)** Young-adult song syllables are significantly more variable than those of older adults (*n* = 34 syllables from young-adults; *n* = 102 syllables from older adults; asterisk *p* < 0.05 Komolgorov-Smirnov test).

Consistent with prior findings in both songbirds and humans, we found that vocal variability decreases with age in the months following the initial acquisition of vocal behavior (Lee et al., 1999; Deregnaucourt et al., 2005; Gerosa et al., 2007; Olveczky et al., 2011). We collected data from two groups of male Bengalese finches: “young-adults” (*n* = 13; mean age 106 days post-hatch, or “dph,” at pitch shift onset) and “adults” (*n* = 11; mean age 377 dph at shift onset). Figure 2A shows a timeline of song development. After a process of developmental song acquisition, the acoustic structure and sequencing of song syllables becomes established (or “crystallized”) when birds reach sexual maturity at ~90 days of age. The similarity between young-adult and adult song can be seen in Figures 2B,C, which show a typical example of young-adult Bengalese finch song just after song acquisition (Figure 2B; 103 dph) and song recorded in the same animal later in adulthood (Figure 2C, 353 dph). Note that although this example illustrates the song of a single animal at two ages, individual animals were only exposed to pitch shifts either as young-adults or as adults (i.e., the groups are non-overlapping populations). We quantified age-dependent differences in vocal variability by using a nonparametric measure of dispersion (median absolute deviation, or MAD) in the pitch of individual syllables (Figure 2D). Comparison of young-adult and adult data (Figure 2E) revealed a significant age-dependent decrease in variability.

### The same sensory error evokes faster and greater vocal changes in younger adults

**Figure 3:**
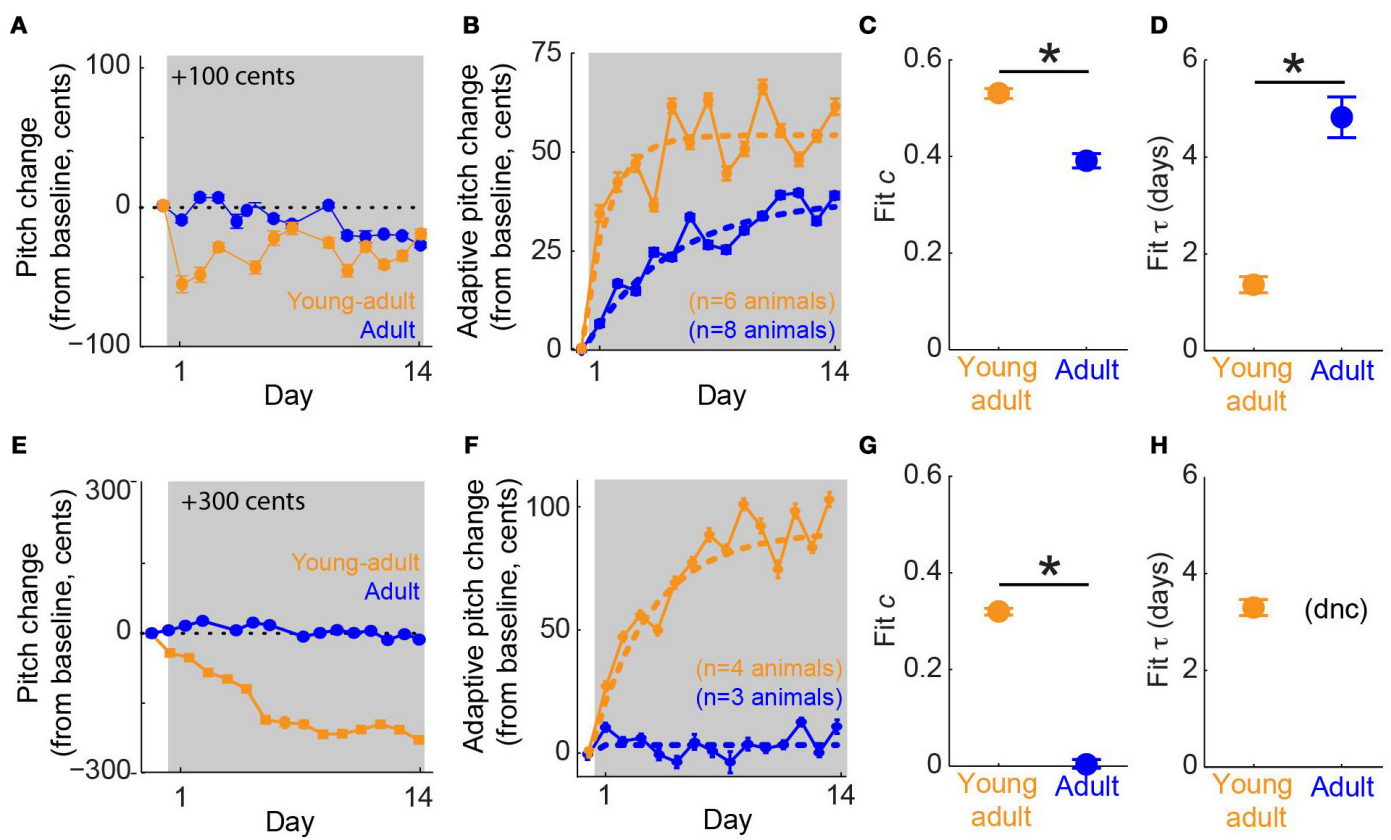
**Sensorimotor adaptation in young-adults is significantly faster and greater in magnitude than adaptation in older adults. (A)** Changes in vocal pitch in response to a 100-cent upward shift in the pitch of auditory feedback for a single experiment in a young-adult songbird (orange, 106 days at shift onset) and a separate experiment in an adult (blue, 384 dph at shift onset). **(B)** Combined data for all ±100 cent shifts, reoriented so that vocal changes in the adaptive direction (opposite to the imposed pitch shift) are positive. Dashed lines; model fits from the standard exponential model (see Methods). Color conventions as in **(A)**. **(C,D)** Fit values from the standard exponential model of free parameters *c*, which quantifies the fraction of the sensory error corrected when sensorimotor adaptation reaches equilibrium and τ, which quantifies the time constant of adaptive behavioral changes. In **(A,B)** error bars show s.e.m. and are sometimes obscured by symbols. In **(C,D)** error bars represent 95% confidence intervals of model fits; asterisks indicate significant differences (*p* < 0.001, permutation test; see Methods). **(E–H)** follow the same conventions as **(A–D)** but show data from experiments using ±300 cent pitch shifts, including representative examples from two birds **(E)**, combined data across all ± 300 cent shifts **(F)**, and model fits **(G,H)**. Note that for ±300 cent pitch shifts in adult animals, the exponential model did not converge (dnc) on a value of τ, as reported previously (Sober and Brainard, 2012).

We tested our hypothesis that young-adult birds would exhibit greater error correction than adults by subjecting both groups of animals to online pitch shifts delivered via miniature headphones (Figure 1A). As described in Methods, the headphones served to replace the bird's natural auditory feedback with a pitch-shifted version. Additionally, because pitch shifts were always performed relative to the current vocal output, birds could reduce or eliminate the experienced auditory error by modifying their vocalizations (Figure 1B). Online pitch shifts reliably led to adaptive changes in vocal pitch (i.e., changes in vocal pitch in the opposite direction of the imposed pitch shift). Consistent with our hypothesis, we found that vocal error correction in young-adult songbirds was significantly more rapid and greater in magnitude than that seen in adults. Figure 3A shows data from two representative experiments, one in a young- adult animal (orange) and one in an adult (blue), both employing +100 cent (upward) shifts in feedback pitch. In these experiments, the young-adult songbird exhibited adaptive vocal changes that were both more rapid in onset and achieved a greater absolute magnitude over most of the shift epoch. Figure 3B shows combined data across all experiments employing ±100 cent shifts. Here, data from upward and downward shifts are combined and reoriented so that adaptive changes in vocal pitch are positive on the vertical axis. These combined data show that on average, young-adult birds exposed to ±100 cent shifts generate faster and greater vocal changes than do older adults experiencing the same error. Analysis of data from ±300 cent shift experiments revealed a similar pattern. Figure 3E shows two representative experiments employing +300 cent (upward) pitch shifts. Although the young-adult bird generated a robust adaptive change in vocal pitch (Figure 3E, orange), the adult bird exhibited no change in vocal pitch by the end of the shift epoch (Figure 3E, blue). Combining data across all ±300 cent shift experiments (Figure 3F) similarly reveals that whereas young-adult birds generate a significant corrective response, learning in older adults is approximately zero. In all four groups (young-adult and adult songbirds undergoing ±100 and ±300 cent shifts) there were no significant differences in adaptation magnitude between upward and downward pitch shifts (*p* > 0.08 in all cases, 2-tailed *t*-tests), as reported previously for adult songbirds (Sober and Brainard, 2009).

To quantify the dynamics of error correction, we fit data from each group with an exponential model of error correction (“standard exponential model”; Equation 1). This model fits two free parameters to the data: *c*, which quantifies the fraction of the imposed pitch shift corrected when adaptation reaches equilibrium, and τ, the time constant of adaptation. Model fits to data from ±100 cent shifts are shown as dashed lines in Figure 3B. Permutation tests (see Methods) revealed significant differences in both *c* and τ across experimental groups (asterisks, Figures 3C,D). Fit values of *c* (0.52, young-adult; 0.40, adult) show that for the same sensory error magnitude (±100 cents), young-adult songbirds correct more than half of the imposed sensory error, whereas older adults correct only ~40% of the error at equilibrium. Furthermore, fit values of τ (1.40 days, young-adult; 4.61 days, adult) show that young-adult songbirds correct 100 cent errors more than three times more quickly than adults.

Model fits to data from experiments involving ±300 cent shifts (dashed lines, Figure 3F) revealed an even larger age-dependent difference in the extent of error correction. Here, fit values of *c* (0.32, young-adult; 0.02, adult; Figure 3G) show that whereas young-adults correct roughly a third of the ±300 cent sensory error, older adults produce no significant adaptive response (the fit value of *c* = 0.02 for adult animals is not significantly different from zero). Therefore, for both ±100 and ±300 cent shifts (Figures 3C,G, respectively), young-adult birds correct a significantly greater fraction of auditory errors than do adult birds. The fit value of τ was 3.47 days for young-adults undergoing ±300 cent shifts (Figure 3H). However, the model did not converge on a value of τ for adult subjects in this condition, likely because the day-to-day pitch fluctuations that take place even in the absence of experimental pitch shifts (Sober and Brainard, 2009) were larger than any adaptive vocal changes, preventing a meaningful measure of the timecourse of error correction. Therefore, although error correction is significantly more rapid in young-adult birds exposed to ±100 cent errors than in adult birds experiencing the same error (Figure 3D), we were unable to compare the value of τ across ages in the ±300 cent shift condition. Finally, we note that in young-adult animals, the fit value of τ during ±100 cent shifts (Figure 3D, orange symbol) was significantly smaller than the fit value of τ during ±300 cent shifts (Figure 3H, orange symbol), consistent with our earlier study demonstrating that the time constant of learning is smaller (i.e., adaptation is more rapid) for smaller error sizes in older birds (Sober and Brainard, 2012).

If young-adult songbirds produced a much larger amount of song each day than did adults, the faster time constant of adaptation (τ) observed in young-adult animals undergoing ±100 cent pitch shifts (Figure 3D) might reflect an increased amount of vocal practice each day, rather than a true difference in adaptation dynamics. We therefore analyzed the data from ±100 cent shifts using an alternate model in which adaptation is quantified in terms of the total number of song syllables produced, rather than time. Although there was substantial subject-by-subject variability in the amount of song production, on average young-adult birds sang more syllables each day than adults (young-adult, mean 12,759 syllables/day, range 5352–23,408; adult, mean 6508 syllables/day, range 1376–13,444). We therefore fit an alternate version of the standard exponential model (Equation 2; Methods) in which adaptation rate is quantified over the number of syllables produced, rather than time in days. This alternate analysis yielded qualitatively the same result as the primary analysis, with fit *c* significantly greater in young-adults and fit τ significantly greater in adults (fit values of [c τ] = [0.51 1.5 × 10^4^ syllables], young-adults; [0.41 2.7 × 10^4^ syllables], adults; differences significant at *p* < 0.001, permutation tests). These results indicate that differences in the amount of song produced do not account for differences in the time constant of error correction shown in Figure 3D. Additionally, all other results reported below were qualitatively identical when the alternate model (Equation 2) was used in place of the standard exponential model (Equation 1).

**Figure 4:**
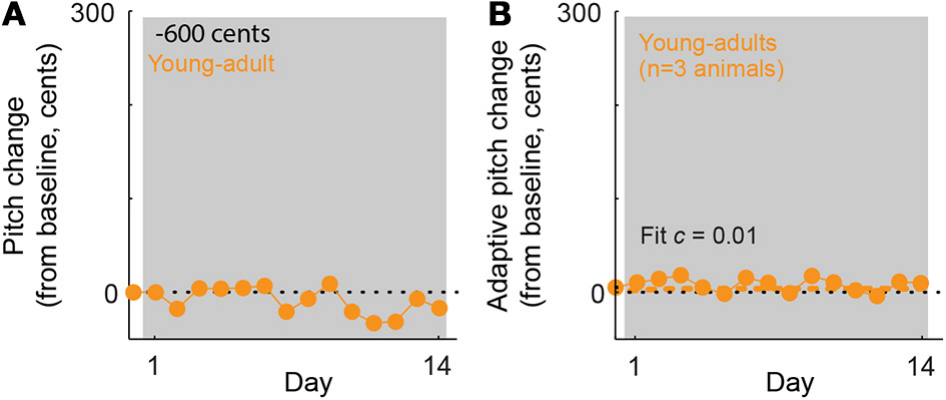
**In young-adult animals, ±600-cent shifts do not evoke significant vocal adaptation. (A)** Changes in vocal pitch in response to a 600-cent downward shift in the pitch of auditory feedback for a single experiment in a young-adult songbird (108 days at shift onset). **(B)** Combined data for all ±600 cent shifts, reoriented so that vocal changes in the adaptive direction (opposite to the imposed pitch shift) are positive. Dashed line; model fit from the standard exponential model (see Methods). The fit value of *c* = 0.01 is not significantly different from zero. Note that for ±600 cent pitch shifts in young-adults, the exponential model did not converge on a value of τ. Other plotting conventions as in Figure 3.

Our results demonstrate that in young-adult songbirds, the fraction of error correction (*c*) decreases as the magnitude of the pitch shift increases (fit *c* = 0.52 for ±100-cent shifts in young-adults, orange symbol in Figure 3C vs *c* = 0.32 for ±300-cent shifts in young-adults, orange symbols in Figure 3G), as reported previously in adult birds (Sober and Brainard, 2012). However, although the fraction of the sensory error corrected declines with increasing sensory error size in young-adults, the absolute magnitude of vocal changes on the last day of the pitch shift is greater in ±300-cent shift experiments than in ±100-cent shift experiments (compare the final value of the orange traces in Figures 3B,F). This pattern of increases in the absolute magnitude of vocal changes as the fraction of compensation decreases was previously shown in adult birds, however in adult birds both the the absolute compensation and the fraction of error correction approached zero once pitch shifts became so large that the overlap between prior and experienced feedback approached zero (Sober and Brainard, 2012). To determine whether the magnitude of error correction similarly approaches zero for larger shift sizes in young-adults, we therefore performed experiments in which young-adult birds were exposed to ±600 cent shifts (data from a representative experiment are shown in Figure 4A). As expected from our prior results in adults, this manipulation led to near-zero learning (Figure 4B; fit *c* = 0.01 was not significantly different from zero), confirming that both the fraction of error correction (*c*) and the absolute magnitude of vocal changes approach zero when sensory feedback is shifted outside of the prior distribution, even if for some error sizes the absolute magnitude of vocal changes can increase with increasing sensory errors.

### Sensory overlap predicts adaptation dynamics across age and error size

**Figure 5:**
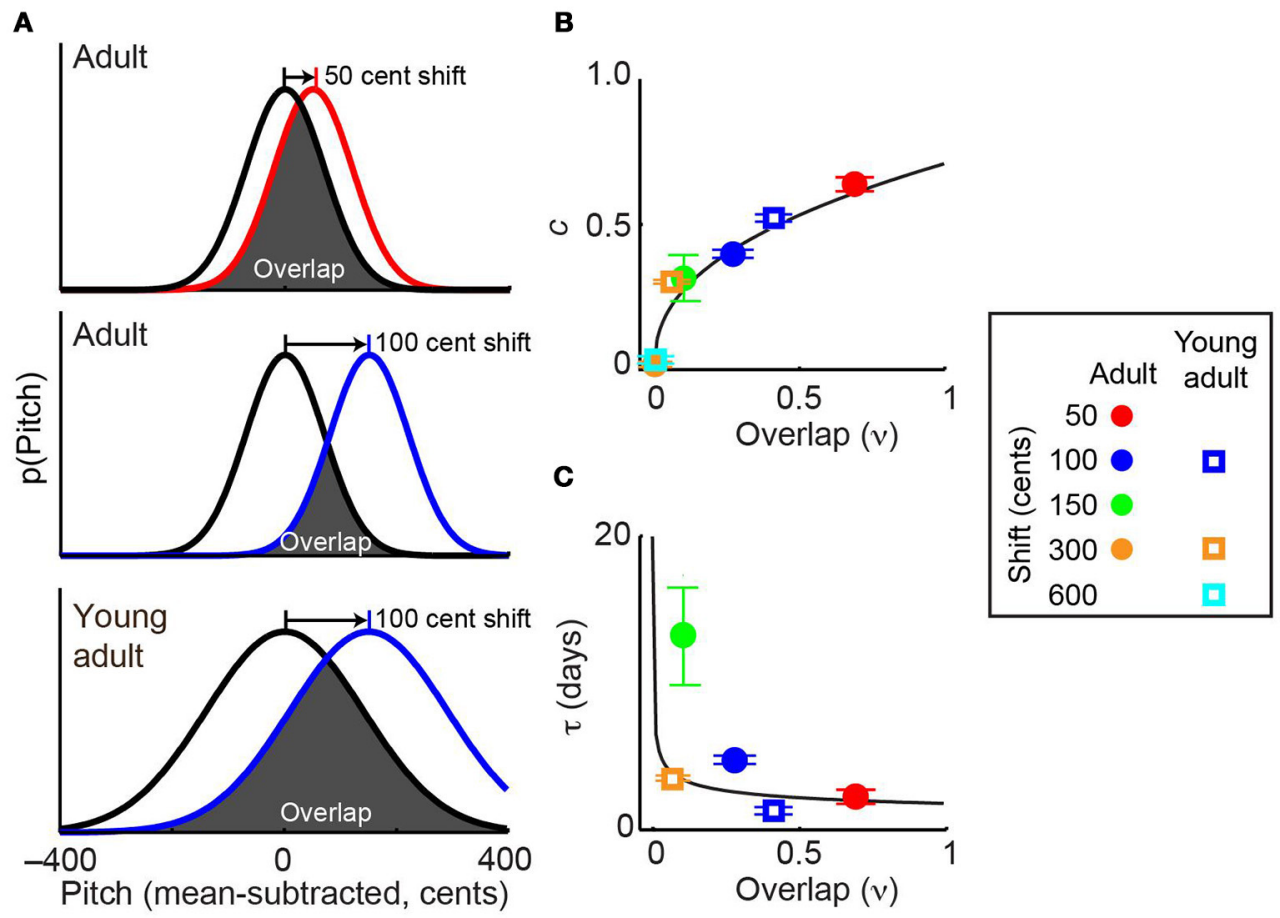
**Overlap with prior distribution predicts the dynamics of sensorimotor adaptation. (A)** Schematics show the overlap between baseline pitch distributions (black) and those experienced at the beginning of pitch shifts (colors). Adults (top two panels) have relatively low pitch variability. Because young-adults have higher variability, a given shift size (e.g., 100 cents) in a young-adult (bottom) will result in greater overlap than the same shift size in an older adult (middle). In this schematic, differences in adult and young-adult variability (Figure 1E) are exaggerated for visual clarity; the actual distributions for each individual subject are shown in Supplemental Figure 1. **(B,C)** The prior overlap model (see Methods) predicts adaptation dynamics across experimental conditions. When fit to the entire dataset, this model uses the overlap shown in **(A)** to predict the fit values of *c*, which quantifies the magnitude of adaptation and τ, the time constant of adaptation. Model predictions of *c* and τ are shown as black traces in **(B,C)**, respectively. Values of *c* and τ in each experimental condition were obtained by fitting the standard exponential model (Equation 1) to each dataset. The standard exponential model did not converge on a value of τ for the 300-cent data in adult subjects or the 600-cent data in young-adults (see text). Error bars in **(B)** and **(C)** indicate 95% confidence intervals and in some cases are partially obscured by symbols. Inset at right shows color and symbol conventions. Note that symbols representing ±300-cent shifts in adults (orange circle) is partially obscured by the symbol representing ±600-cent shifts in young-adults (cyan square) in **(B)**.

Combining the above results with data from experiments employing other shift magnitudes allowed us to test the hypothesis that distribution overlap, rather than absolute error size, is the best predictor of the dynamics of sensorimotor adaptation. Previously (Sober and Brainard, 2012), we demonstrated that in adult songbirds the magnitude of adaptation approaches zero when pitch shifts become sufficiently large that the distribution of pitches heard by the animal (Figure 5A, colored lines) no longer overlaps with the baseline, or prior, distribution (Figure 5A, black lines). Similarly, in other systems the magnitude of error correction decreases as sensory error size increases (Robinson et al., 2003; Fine and Thoroughman, 2007; Wei and Kording, 2009; Marko et al., 2012). While these previous findings suggest that distribution overlap (ν, see Methods) might be an important determinant of the magnitude of error correction at equilibrium, it is unclear whether the reported differences in adaptation reflect differences in overlap or simply in the absolute magnitude of the sensory error itself. Similarly, it is unknown whether overlap may be able to account for the age-dependent changes in error correction shown in Figure 3. Because young-adult song is more variable than adult song (Figure 2E), a given shift magnitude will result in greater overlap in young-adults (Figure 5A, middle and bottom panels), thereby dissociating shift size and overlap and allowing us to investigate which factor better predicts the magnitude of sensorimotor adaptation.

Differences in vocal plasticity across age groups and error sizes were well predicted by a model that uses the overlap between prior and experienced feedback (“prior overlap model,” see Methods) to predict the dynamics of sensorimotor adaption. Figures 5B,C shows that the best-fit prior overlap model (black lines) predicts adaptation parameters *c* and τ across experimental conditions. Note that the prior overlap model is fit to all individual pitch measurements recorded in our experiments, not to the values of *c* and τ shown as colored symbols in Figures 5B,C.

As described in Methods, we evaluated the predictive power of the prior overlap model by both comparing it to an alternate model of sensorimotor adaptation and asking whether the relationship between overlap and error correction observed in the adult data could predict the results of experiments in young-adults. First, we found that the prior overlap model explains a significantly greater amount of the total data variance than the standard exponential model (*F*-test, *p* < 0.001), indicating that the increased predictive power of the overlap model is significantly greater than that expected by chance given that this model includes one more free parameter than the exponential model (see Methods). This result demonstrates that a model based on the overlap between prior and experienced sensory feedback provides a better prediction of adaptation dynamics than one that predicts learning based on sensory error size. Second, we asked whether fitting the prior overlap model to data from adults provided a good prediction of data from young-adults. To do so, we tested whether fitting each free parameter in the prior overlap model (α_*c*_, α_τ_, and ρ; Equation 3) separately to the young-adult data provided a significant increase in predictive power. This was not the case (*F*-test for inclusion of separate predictor terms for the young-adult data was not significant, *p* > 0.9 for all three tests), demonstrating that young-adult data were well predicted based on fitting the prior overlap model to the adult data.

## Discussion

Our results demonstrate that in adult songbirds with crystallized songs, the rate and magnitude of vocal error correction decrease significantly between early adulthood and later adulthood and the overlap of prior and experienced sensory feedback predicts the dynamics of error correction across a range of experimental conditions. When young-adult and adult songbirds were subjected to online pitch shifts of the same magnitude (±100 or ±300 cents), we found that young-adult birds corrected a greater fraction of the error (*c*) and did so more rapidly (τ) than adults (Figure 3). Furthermore, we found that the dynamics of learning were better explained by a model that predicts error correction based on the overlap between the animals' baseline pitch distribution and that experienced following a pitch shift (Figure 5) than by a model that predicts learning solely based on the sensory error.

Our analysis shows that the overlap between prior and experienced sensory feedback predicts the extent of sensorimotor adaptation across a variety of experimental conditions. Studies in a wide range of species and behaviors have shown that the dynamics of error correction can vary significantly with the size of the sensory error and the age of the subject (Robinson et al., 2003; Contreras-Vidal et al., 2005; Fine and Thoroughman, 2007; Wei and Kording, 2009; Shiller et al., 2010; Liu et al., 2010b, 2011). The predictive power of the prior overlap model (Figures 5B,C; black traces) therefore suggests that the age- and error size-dependent learning differences observed in songbirds (and possibly other behaviors and species as well) reflect a consistent relationship between adaptation dynamics and the statistics of prior and experienced sensory feedback. Future studies might test this hypothesis by manipulating the variability of experienced auditory feedback, for example by reducing feedback variability to adult levels in a young-adult animal or increasing the variability experienced by an adult. Such experiments could confirm the role of prior sensory experience in determining learning dynamics by dissociating it from the age of the subject.

Our results demonstrate that Bengalese finches that have recently acquired their songs exhibit significantly faster and more complete error correction than animals tested several months later in adulthood (Figure 3). Although the basic acoustic structure of Bengalese finch song syllables crystallizes at ~90 dph (Clayton, 1987), prior studies have described consistent differences in diurnal acoustic changes (Deregnaucourt et al., 2005) and song tempo (Arnold, 1975; Glaze and Troyer, 2013) between young-adult and adult animals, in each case accompanied by an age-dependent drop in variability. Additionally, Bengalese finches in some cases exhibit changes in syllable sequencing after 90 days (Lipkind et al., 2013). Pitch is one of several parameters that are finely tuned during the process of song acquisition (Tchernichovski et al., 2001), and our demonstration that the same pitch error evokes greater adaptive vocal changes in young-adult animals compared to older adults suggest that a similar mechanism is used to refine other vocal parameters in young-adults. Future studies might also investigate whether the rate and magnitude of error correction is even greater in birds younger than 90 days, who have not yet crystallized their songs (Figure 2A). Although such measurements are not possible with the present experimental technique—our quantitative analysis (see Methods) relies on repeated measurements of the pitch of identifiable song syllables, and we were unable to reliably identify individual song syllables in Bengalese finches younger than 90 days—future studies employing other means to measure adaptation in juvenile birds might make this possible.

Studies of the songbird brain suggest a potential neural substrate for age-dependent changes in variability. The lateral magnocellular nucleus of the anterior nidopallium (LMAN), the output nucleus of a basal ganglia pathway, plays a crucial role in both the generation of vocal variability and in vocal plasticity. Production of highly variable “subsong” early in development (much earlier than the ages of the young-adult birds tested here) is abolished by LMAN inactivation, whereas lesions of LMAN later in life reduce the variability of (but do not eliminate) song (Kao et al., 2005; Olveczky et al., 2005; Aronov et al., 2008). Therefore, although age-dependent differences in the influence of LMAN between young-adult and adult songbirds have not been fully characterized, it is plausible that the observed age-dependent reduction in variability (Figure 2E) might reflect a further decline in the influence of LMAN on vocal production. A number of studies have suggested that a reinforcement learning mechanism guides vocal plasticity by evaluating variations in vocal output driven by LMAN (Andalman and Fee, 2009; Fee and Goldberg, 2011; Warren et al., 2011) or other components of the song system (Charlesworth et al., 2012). Although our behavioral paradigm does not include explicit reward or punishment signals, internal reinforcement signals might be activated when the pitch of experienced auditory feedback falls within the baseline pitch distribution. In this model, smaller pitch shifts would result in a greater number of reinforced trials—and thus greater learning—because of the greater overlap between the baseline and shifted distribution (Figure 5A, top). Since age-dependent changes in variability, perhaps resulting from changes in LMAN's influence on vocal output, result in a narrowing of the baseline pitch distribution, the reduced learning in older animals might reflect a reduced number of rewarded trials for a given magnitude of sensory errors. Additionally, practice-dependent effects (motor “engrainment”) might operate independently of the basal ganglia circuitry to reduce variability, and thereby reduce learning, as the animal ages (Lombardino and Nottebohm, 2000).

Additionally, our data from songbirds parallel similar results in studies of human vocal control. For example, in response to ±100 cent pitch shifts imposed during singing, human subjects with greater vocal variability compensate more than subjects with lesser variability (Scheerer and Jones, 2012), consistent with our model (Figure 5). Similarly, in response to online pitch perturbations, children, who exhibit greater pitch variability than adolescents or adults (Lee et al., 1999; Walsh and Smith, 2002), exhibit greater compensatory responses (Liu et al., 2010b). Therefore, although the presence and magnitude of age-dependent changes in vocal error correction in humans vary somewhat across different studies (Liu et al., 2010a,b; Shiller et al., 2010; Macdonald et al., 2012)—which themselves vary in terms of experimental design and the age of subjects—multiple studies in adults and children suggest that variability is a strong predictor of vocal error correction in humans as well as songbirds, potentially reflecting a general principle of learned vocal behaviors.

Constraining sensorimotor adaptation based on sensory overlap (Figure 5) is a robust method for maintaining the accuracy of complex, learned behaviors. Error correction necessarily falls between the extremes of adaptability and stability. A highly adaptable system can generate rapid corrections but risks destabilization if sensory feedback is contaminated by noise in the nervous system or the environment (Faisal et al., 2008). Emphasizing stability, in contrast, reduces the vulnerability to aberrant sensory signals but also reduces the ability to correct legitimate errors. Allowing greater adaptability when performance is poor but reducing adaptability after performance improves could therefore maximize both learning rate and post-learning performance.

Our data suggest that the brain balances adaptability and stability by correcting apparent sensory errors when they overlap with the probability distribution of previously experienced sensory feedback but ignoring larger errors. Such a strategy has two key advantages. First, it uses the statistics of the animal's own sensory experience (rather than, for example, an explicit model of sensory noise) to evaluate whether and how fast to correct an apparent error. Such an approach could improve behavioral performance without requiring the brain to explicitly calculate the probability of error relevance (Wei and Kording, 2009) or the statistics of the extrinsic sensory perturbations (Fine and Thoroughman, 2007). Second, such an approach will stabilize behavior as performance improves. When a behavior becomes less variable, overlap—and therefore the predicted learning—in response to a given error size will decrease, as shown in our comparison of adult and young-adult data. Therefore, the computational principle described by the prior overlap model allows both greater adaptability shortly after acquisition of a new skill, when behavior is less precise, and greater stability later on, when performance variability decreases. Applying the analyses presented here to adaptation data collected in other systems could reveal whether the simple and robust computational strategy we describe is employed to modify non-vocal behaviors as well.

### Conflict of interest statement

The authors declare that the research was conducted in the absence of any commercial or financial relationships that could be construed as a potential conflict of interest.
