# Supplementary figures and images for: A simple computational principle predicts vocal adaptation dynamics across age and error size
Source: Front Integr Neurosci. 2014 Sep 29;8:75. doi: 10.3389/fnint.2014.00075 (PMC4178380; doi:10.3389/fnint.2014.00075)

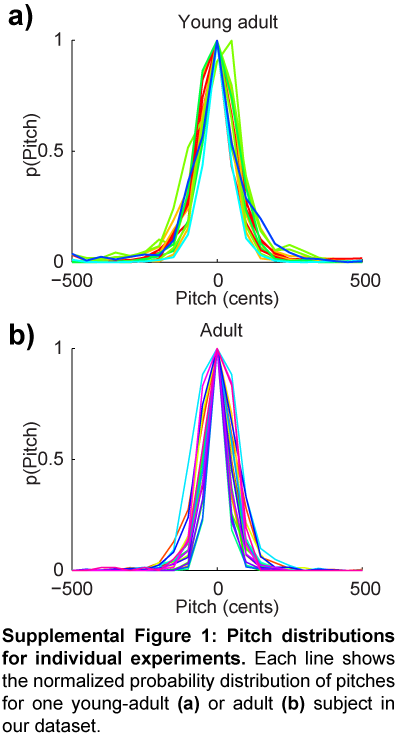

Supplement: Supplementary file 1 [file Image1.TIF]
